# Correction of Background in Fluorescence Correlation Spectroscopy for Accurate Determination of Particle Number

**DOI:** 10.3390/biom16010011

**Published:** 2025-12-20

**Authors:** Elisa Longo, Greta Paternò, Elisabetta Di Franco, Paolo Bianchini, Marco Castello, Alberto Diaspro, Giuseppe Vicidomini, Elena Bruno, Paolo Musumeci, Maria Josè Lo Faro, Nunzio Tuccitto, Luca Lanzanò

**Affiliations:** 1 Department of Physics and Astronomy “Ettore Majorana”, University of Catania, 95123 Catania, Italygreta.paterno@unict.it (G.P.); elisabetta.difranco@edu.unige.it (E.D.F.); paolo.musumeci@dfa.unict.it (P.M.); 2 Nanoscopy, CHT Erzelli, Istituto Italiano di Tecnologia, 16163 Genoa, Italy; 3 R&D Department, Genoa Instruments S.r.l., Via E. Melen 83, 16152 Genova, Italy; marco.castello@genoainstruments.com; 4 DIFILAB, Department of Physics, University of Genoa, 16126 Genoa, Italy; 5 Molecular Microscopy and Spectroscopy, CHT Erzelli, Istituto Italiano di Tecnologia, 16163 Genoa, Italy; 6 Consiglio Nazionale delle Ricerche-Istituto per la Microelettronica e Microsistemi (CNR-IMM), Catania-Unit, Via Santa Sofia 64, 95123 Catania, Italy; 7 Department of Chemical Sciences, University of Catania, 95125 Catania, Italy; nunzio.tuccitto@unict.it; 8 Istituto Nazionale di Fisica Nucleare (INFN), Sezione di Catania, Via S. Sofia 64, 95123 Catania, Italy; 9 Centro Siciliano di Fisica Nucleare e Struttura della Materia-CSFNSM, 95123 Catania, Italy

**Keywords:** FCS, uncorrelated background, confocal microscope, carbon dots, SPAD array

## Abstract

Since the early development of Fluorescence Correlation Spectroscopy (FCS), it has been recognized that background intensity can lead to artifacts in the amplitude of the autocorrelation function (ACF) and, consequently, to inaccurate estimates of particle numbers. Here, we present a protocol for quantitative background evaluation and amplitude correction in FCS experiments, applicable to different sources of background such as detector noise, autofluorescence, and light scattering. We demonstrate the performance of our approach through three representative case studies: (i) FCS measurements of a bright fluorophore at low concentration, (ii) FCS of dim nanoparticles affected by solvent Raman scattering, and (iii) FCS performed using a confocal setup equipped with a SPAD array, where background originates from detector hot pixels. These examples represent typical experimental conditions in which background signals compromise quantitative interpretation, illustrating how our protocol restores accuracy and reproducibility in FCS analysis. By systematically identifying and correcting these effects, the proposed protocol addresses a long-standing limitation of FCS and provides a robust framework for improving the accuracy and reproducibility of quantitative fluorescence measurements.

## 1. Introduction

Fluorescence Correlation Spectroscopy (FCS) is a powerful and versatile technique for the quantitative analysis of molecular dynamics, widely applied in fields such as biomedicine, biophysics, and chemistry [1]. Originally introduced in 1972 by Magde et al. to study the binding of the fluorescent dye ethidium bromide to DNA [2], FCS has since evolved into a key tool for investigating molecular diffusion and particle quantification and interactions [3]. A major advancement came in the 1990s, when FCS was integrated with confocal microscopy. This allowed the excitation and detection volumes to be confined to the femtoliter scale (∼0.5 fL), significantly enhancing the signal-to-noise ratio and enabling sensitive detection of fluorescence fluctuations in highly localized regions [4].

FCS analyzes the spontaneous fluctuations in fluorescence intensity caused by the random motion of fluorescent molecules through the observation volume. These fluctuations are characterized through the autocorrelation function (ACF), whose width depends on the diffusion coefficient of the particles and whose amplitude depends on the number of particles N in the observation volume. Indeed, from the autocorrelation of the molecule fluorescence temporal fluctuations induced by focused radiation, FCS can quantitatively evaluate the concentration, diffusion coefficient, hydrodynamic radius, and target interactions in different matrices [5,6]. To maximize sensitivity and reduce background contributions, FCS is typically performed at low concentrations, ensuring a small number of fluorescent molecules in the detection volume, which is usually defined by a high numerical aperture objective in a confocal microscope.

When applied to complex biological systems, however, FCS meets several challenges, including, for instance, cellular motion, photobleaching of fluorophores, and the complexity of anomalous modes of diffusion. Over the past two decades, several methodological advances have addressed these challenges. Dual-color Fluorescence Cross-Correlation Spectroscopy (FCCS) enables the study of molecular interactions [7]. Pair Correlation Function (pCF) analysis provides information on directional transport between spatially separated locations [8,9]. Light-sheet-based FCS (SPIM-FCS) combines selective plane illumination with correlation analysis, simultaneously acquiring information from thousands of pixels while minimizing out-of-focus contributions [10]. Parallelized FCS, based on detector arrays or fast cameras, allows simultaneous acquisition of fluctuations across multiple regions [11]. Spot variation FCS, often combined with super-resolution techniques such as stimulated emission depletion (STED), allows the investigation of diffusion processes at different spatial scales [12,13]. Scanning FCS approaches, employing fast line or circular scans, allow the generation of spatial diffusion maps or computation of pairwise correlations across pixels [9,14,15]. Among these developments, the segmented FCS approach introduced by Di Bona et al. [16] divides continuous scan data into short segments, treating each as an independent FCS measurement; by sorting and averaging the ACFs, it enables simultaneous analysis of molecular dynamics in different regions from a single acquisition. Subsequent studies, such as those by Kohler et al. [17], have explored the impact of segmentation on fitting accuracy, particularly when using very short time windows. These advancements reflect the continuous evolution of FCS methodologies, which now include a wide range of experimental configurations with different spatial and temporal scales.

A long-standing problem in FCS, common to early developments and modern applications of the technique, is the presence of background noise. Since its early developments, it was recognized that the presence of background intensity can strongly influence the outcome of FCS experiments, leading to distortions in the amplitude of the autocorrelation function and consequently in the estimation of particle numbers and diffusion parameters [18]. In general, background fluctuations are not autocorrelated at the timescales of interest (hence the name ‘uncorrelated’ background), but a significant level of background intensity may artificially reduce the correlation amplitude and thereby bias quantitative results [18].

Several sources can contribute to uncorrelated background signals, including autofluorescence, scattering, and detector noise [19,20]. In these cases, the impact of background on FCS measurements becomes even more critical at low fluorophore concentrations or for low efficiency emitters, where the relative contribution of background photons to the overall signal is higher. Indeed, the measured intensity can be considered as the sum of the desired molecular fluctuations, F(t), which depends linearly on the concentration and brightness of the fluorophores, and the background contribution, B(t), independent from the concentration of fluorophores. When the fluorophore concentration is low, the relative contribution of B(t) is higher.

In other cases, the background can originate from the same fluorophores that generate the fluctuations. For instance, in TIRF-FCS, background can be generated from labeled ligand freely diffusing through the detection volume on a much shorter timescale than the considered binding kinetics [21]. In STED-FCS, fluorescence depletion should result in a reduction in both the average molecular transit time and the number of detected molecules with increasing STED laser intensity. In practice, however, experiments in 3D diffusion conditions have shown that while transit times decrease as expected, the apparent number of molecules does not, due to the presence of low-brightness fluorescence from undepleted out-of-focus regions [22,23,24,25]. Several strategies have been proposed to remove background in STED-FCS based on double depletion [24], polarization switching [26], and lifetime separation [22].

In general, accurate background estimation and correction are crucial to obtain reliable molecular concentrations and diffusion parameters, as extensively discussed in previous works addressing the effects of noise and photobleaching in FCS measurements [27] and the comparison between different background correction approaches [28].

Here, we discuss a protocol for evaluating the background in FCS experiments and for correcting the amplitude of the ACF. This protocol will work whenever the background contribution is not related to the fluorophores that generate the fluctuations but is generated by another source, for instance detector noise, autofluorescence, and scattering. If this is the case, one can easily determine the contribution of the background by measuring a sample without the fluorophore (unlabeled sample) and then correct the amplitude of the ACF. We integrate this background correction in a recently developed software that performs segmented FCS using data acquired on a laser scanning microscope (LSM) [29]. We have demonstrated that segmented FCS can be implemented on a commercial LSM and can be used to measure mobility of fluorophores in solution and in different subcellular regions [29,30]. We test this approach on three case studies: (i) FCS of a bright fluorophore at low concentration, (ii) FCS of dim nanoparticles, where we see that part of the background originates from Raman scattering of the solvent, and (iii) FCS using a confocal setup equipped with a SPAD array, where background arises from hot pixels of the detectors. These examples reflect common situations encountered in both fundamental and applied FCS studies, where background contributions can compromise data interpretation, with particular implications for biological systems. By systematically assessing and correcting for these effects, our protocol addresses an established limitation of FCS and provides a practical strategy to improve the robustness of quantitative measurements.

## 2. Materials and Methods

### 2.1. Generation of a Background-Corrected Correlation Function

In ideal FCS measurements, fluctuations in fluorescence intensity originate from the fluctuations in the number of fluorescent particles in the observation volume, and the amplitude of the autocorrelation function (ACF) is directly proportional to the inverse of the average particle number (Figure 1a, left). In the presence of background, the relative amplitude of the fluctuations is smaller and the amplitude of the ACF decreases (Figure 1a, right).

The intensity contains a contribution due to the fluorescent particles I_P_(t) and a contribution due to the background I_B_(t):

(1) $$I\left(t\right)=I_{P}\left(t\right)+I_{B}\left(t\right)$$

The autocorrelation function (ACF) of the intensity I(t) is defined as:

(2) $$G\left(\tau \right)=\frac{\left\langle I\left(t\right)I\left(t+\tau \right)\right\rangle }{{\left\langle I\left(t\right)\right\rangle }^{2}}-1$$

where the brackets indicate averaging over time. In particular, for the time lag τ = 0:

(3) $$G\left(0\right)=\frac{\left\langle {I(t)}^{2}\right\rangle }{{\left\langle I\left(t\right)\right\rangle }^{2}}-1=\frac{\left\langle {I\left(t\right)}^{2}\right\rangle -{\left\langle I\left(t\right)\right\rangle }^{2}}{{\left\langle I\left(t\right)\right\rangle }^{2}}=\frac{\sigma^{2}}{{\left\langle I\left(t\right)\right\rangle }^{2}}$$

where σ^2^ is the variance of the intensity and I_av_ = ⟨I(t)⟩ is its average value.

For independent processes, the total variance is the sum of the variances, thus:

(4) $$G\left(0\right)=\frac{\sigma_{P}^{2}+\sigma_{B}^{2}}{{\left(I_{av,P}+I_{av,B}\right)}^{2}}$$

where σ_P_^2^ and I_av,P_ are the variance and average of I_P_(t), respectively, and σ_B_^2^ and I_av,B_ are the variance and average of I_B_(t), respectively. If we assume that the correlation of the background is negligible (uncorrelated background), then σ_B_^2^ = 0 and σ^2^ = σ_P_^2^:

(5) $$G\left(0\right)=\frac{\sigma_{P}^{2}}{{\left(I_{av,P}+I_{av,B}\right)}^{2}}$$

which shows that increasing values of uncorrelated background dampen the amplitude of the ACF (Figure 1a).

We are interested in calculating the amplitude of the ACF of the intensity contribution due to the particles, G_P_ (0):

(6) $$G_{P}\left(0\right)=\frac{\sigma_{P}^{2}}{{\left(I_{av,P}\right)}^{2}}=\frac{\sigma^{2}}{{\left({I_{av}-I}_{av,B}\right)}^{2}}$$

This can be written as:

(7) $$G_{P}\left(0\right)=G\left(0\right)\frac{1}{{\left(1-\frac{I_{av,B}}{I_{av}}\right)}^{2}}=G\left(0\right)\frac{1}{{\left(1-i_{B}\right)}^{2}}$$

where we have defined i_B_ = I_av,B_/I_av_ as the fraction of uncorrelated background.

Equation (7) is a correction formula for the amplitude of the correlation function that requires estimation of the fraction of uncorrelated background. For instance, when the fraction of uncorrelated background is 0.3, the amplitude must be corrected by a factor of 2.

To estimate this fraction and correct the ACF, we follow this step-by-step protocol (Figure 1b):

(1) Perform the measurements on the specimen of interest (‘sample’).
(2) Perform an identical type of measurement (i.e., with the same acquisition parameters such as excitation power and pixel dwell time) on the unlabeled sample (‘background’). If the sample is a solution of fluorophores, the unlabeled sample is the solvent. This measurement will contain several possible sources of background such as detector noise, Raman scattering, and autofluorescence in the conditions of the experiment.
(3) Process the ‘background’ data to extract and save the average background intensity value, I_av,B_.
(4) Process the ‘sample’ data and recall the saved value I_av,B_ to generate the corrected ‘sample’ ACF via the formula reported in Equation (7).

### 2.2. Preparation of Fluorescent Solutions

Solutions of fluorophores were prepared at different concentrations. A higher contribution of background is expected at the lower concentrations. Thus, our protocol is expected to be most effective at the lowest concentrations analyzed. Alexa Fluor 488 Azide (Invitrogen, A10266, Thermofisher Scientific, Waltham, MA, USA) was diluted in PBS at the following dilutions: 1:10^3^, 1:10^4^, 1:10^5^, and 1:10^6^. PARPi-FL (Tocris Bioscience, Bristol, UK, Cat. No. 6461) was reconstituted in DMSO to obtain a 1 mM stock solution. For FCS measurements, PARPi-FL was diluted in water to the final concentrations: 0.001, 0.01, 0.1, and 1 µM. Carbon dots (CDs) employed in this study were prepared as described in [31]. Briefly, CDs were synthesized from citric acid (Merck, Rome, Italy) via a pyrolysis process. Approximately 10 g of citric acid was weighed and heated in a beaker on a hot plate maintained between 200 °C and 250 °C. After 10–15 min, the reaction yielded a dark brown caramel-like product, which was then cooled to room temperature. A 0.2 M NaOH solution (Merck, Italy) was subsequently added until the mixture reached a neutral pH. The resulting suspension underwent membrane dialysis using Membra-Cel MC18 tubing (molecular weight cut-off = 14,000 Da) to remove water-soluble residues and by-products from pyrolysis. The dialysis water was replaced periodically until it became non-fluorescent, typically after about two days. The purified suspension was then subjected to cryo-centrifugation for 2 h at 10,000 rpm and 3 °C. The recovered supernatant was used for further characterization and Molecular Communication (MoCo) experiments. The typical concentration of the as-prepared CD solution was approximately 2 mg mL^−1^. The samples for FCS were prepared by diluting the CDs in water at the indicated dilutions (1:125, 1:250, 1:500, and 1:1000). All solutions were deposited on eight-well Ibidi chambered coverslips (Ibidi, Gräfelfing, Germany, µ-slide 8 well glass bottom, 80821) prior to measurement.

### 2.3. Data Acquisition

Measurements on samples of Alexa 488 Azide and CDs are performed on a Leica TCS SP8 confocal laser scanning microscope (Leica Microsystems, Wetzlar, Germany), using a 1.40 NA 63× objective (HCX PL APO CS2 63/1.40 Oil Leica Microsystems). We used an excitation wavelength of 488 nm (Alexa 488 Azide) or 405 nm (CDs), with emission detection bands of 500–530 nm and 535–600 nm via hybrid detectors operating in photon-counting mode, unless specified otherwise. The use of two detectors enables the removal of the detector afterpulse. FCS data are acquired as XY (raster scan) images in bidirectional slow scanning mode by setting the scanner at a line frequency of 10 Hz and maximum zoom, corresponding to a scanner speed of 154 µm/s and a pixel time of 3.04 µs, as described previously [29].

Spectral imaging is performed using the lambda-scan mode of the Leica TCS SP8 microscope, with emission collected through hybrid detectors operating in photon-counting mode.

Measurements on samples of PARPi-FL are acquired on a SPAD array system (PRISM, Genoa Instrument SRL, Genoa, Italy), implemented as a modification of a confocal laser-scanning microscope with the architecture of an image-scanning microscope [32]. A 1.40 NA 60× objective (PLAN APO λD 60/1.4 Oil Nikon, Amstelveen, The Netherlands) is used for detection. Excitation is performed with a 488 nm laser (QuixX Picosecond-pulsed Diode Laser 488-200 PS 200 mW, Omicron-Laserage Laserprodukte GmbH, Rodgau, Germany) operated in continuous-wave mode. The measurements are achieved with 7 × 7 silicon SPAD array technology. For the FCS analysis, the intensity signal is obtained by integrating over all detector pixels, and the corresponding autocorrelation function is calculated by cross-correlating the signal from odd and even pixels to correct the detector afterpulse contributions and detector-related artifacts. Single-point FCS measurements are performed with a pixel dwell time of 10 µs.

For a given type of sample, the background is evaluated by performing a measurement on the solvent (unlabeled sample) under the very same experimental conditions.

### 2.4. Data Processing and Analysis

The raster scan (XY) data from Leica TCS SP8, saved as .lif files, are opened in ImageJ (https://imagej.net/software/fiji/, accessed on 1 June 2025) and then saved as TIF files. Each file is an image with dimensions N_px_ × N_lines_, with N_px_ = 8192 and N_lines_ = 1024.

Single-point FCS data from the PRISM setup contain 4,000,000 temporal points and 49 detector pixels. The data are saved as .mat files, opened with MATLAB (R2020b, Mathworks, Natick, MA, USA), and exported as TIF files. The 4,000,000 temporal points are reshaped into images of 8000 × 500 pixels. The signal from the 49 detectors is binned into 2 channels containing the sum of odd and even pixels, respectively. The cross-correlation of odd and even pixels is used to remove the detector afterpulse.

The TIF files are opened with a custom script in MATLAB (Mathworks) and processed for the extraction of the autocorrelation function (ACF) with and without correction of background. The script is a modified version of the Segmented FCS software (https://github.com/llanzano/SegmentedFCS, accessed on 1 June 2025) described previously [29]. In this version, the average value of the intensity of the background *I*_*B*,*av*_ is used to correct the amplitude of the ACF. The corrected amplitude of the ACF, G_corr_ (0), is calculated as:

(8) $$G_{corr}\left(0\right)=G_{raw}\left(0\right)\cdot f_{b}$$

where G_raw_(0) is the uncorrected amplitude calculated by the script [29] and f_b_ is the correction factor calculated as:

(9) $$f_{b}=\frac{1}{{\left(1-\frac{I_{B,av}}{I_{av}}\;\right)}^{2}}$$

where I_B,av_ is the average intensity of the background and I_av_ is the average intensity in the sample.

The ACFs are fitted in Origin (OriginLab, Northampton, MA, USA) using the following model, corresponding to diffusion and scanning dynamics:

(10) $$G\left(\tau \right)=G_{diff}\left(\tau \right)\cdot S\left(\tau \right)=G\left(0\right)\cdot \frac{1}{1+\frac{4D\tau \;}{w_{0}^{2}}}\cdot \exp\left(-\frac{{\left(\frac{v\;\tau }{w_{0}}\right)}^{2}}{1+\frac{4D\tau \;}{w_{0}^{2}}}\right)$$

where G(0) is the amplitude of the ACF, D is the diffusion coefficient, w_0_ is 1/e^2^ size of the focal spot in the lateral direction, and v is the speed of the scanner. For single-point FCS data, v = 0.

The number of particles is calculated as:

(11) $$N=\frac{\gamma }{G\left(0\right)}$$

where γ is the gamma factor (γ = 0.35 for a Gaussian PSF in 3D) [22] and G(0) is the amplitude of the ACF.

The concentration is calculated as:

(12) $$C=\frac{N}{V}$$

where V = (π/2)^3/2^w_0_^2^w_z_ is the volume of the confocal detection spot [33], with w_z_ being the size along the axial direction and w_z_ = 0.62 μm.

To analyze the dependence of the calculated number of particles versus the dilution, we plotted the data in log-log scale and performed an apparent linear fit using Origin, so that direct proportionality corresponds to a slope equal to 1. From each fit, we extracted the value of the slope and the corresponding standard error.

## 3. Results

### 3.1. FCS of a Bright Fluorophore at Low Concentration

As a preliminary step, we evaluated background contributions arising from the solvent and instrument noise by measuring the diffusion of Alexa 488 Azide at variable dilutions (from 10^−3^ to 10^−6^) using the Leica TCS SP8 setup. Firstly, we performed FCS on PBS solution only (Figure 2a, top) in order to evaluate the average intensity value of the uncorrelated background (Figure 2a, bottom). At the highest concentration of Alexa 488 Azide, the fluorescent signal dominated over the background (Figure 2b, top), and background correction had minimal impact on the ACF (Figure 2b, bottom). At the lowest concentration, the fluorescent signal was comparable to the background (Figure 2c, top), and background correction increased the amplitude of the ACF (Figure 2c, bottom), showing the need for performing background correction when the concentration of the fluorophore is low. Consistently, the trend of the number of particles (Figure 2d, top) and the measured concentration (Figure 2e) as a function of dilution were closer to directly proportional after background correction, confirming the efficiency of the approach. Indeed, background-corrected data (black) yielded a linear fit with a slope closer to 1 in log-log scale (slope= 0.96 ± 0.05) compared to the uncorrected ones (light blue, slope= 0.8 ± 0.04).

**Figure 2:**
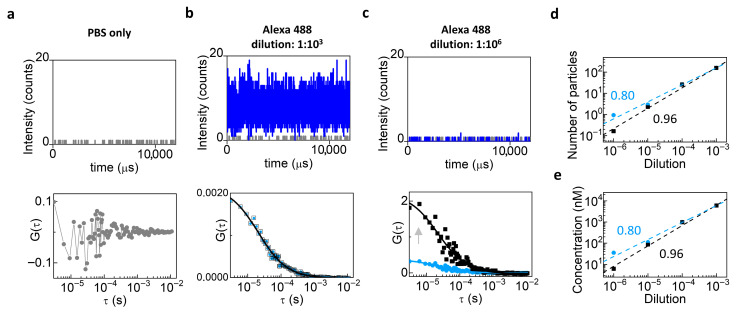
Background correction in FCS measurements of Alexa 488 Azide. (**a**) PBS-only intensity (**top**) and corresponding ACF (**bottom**). (**b**) Alexa Fluor 488 Azide at 10^−3^ dilution: intensity counts (**top**, blue line) and ACFs before (light blue) and after (black) background correction (**bottom**). (**c**) Alexa Fluor 488 Azide at 10^−6^ dilution: intensity counts (**top**, blue line) and ACF showing higher values of G(0) after correction (**bottom**, black) compared to the ACF before correction (**bottom**, light blue). (**d,e**) Number of particles (**d**) and concentration (**e**) as a function of dilution in log-log scale. To check for direct proportionality, we plotted the data in log-log scale and performed an apparent linear fit using Origin. In theory, direct proportionality should correspond to a slope equal to 1. Background-corrected data (black) yield an apparent linear fit with a slope closer to 1 (slope = 0.96) with respect to uncorrected data (light blue, slope = 0.8).

### 3.2. FCS of Dim Nanoparticles

Next, we performed FCS on a sample of fluorescent nanoparticles, specifically carbon dots (CDs) suspended in water at variable dilutions (Figure 3). CDs are nanoparticles mainly based on carbon, plus certain heteroatoms (such as O, N, or S) obtained starting from organic precursors. In general, they exhibit fluorescence in the visible range when excited in the ultraviolet region [34] and their use has been proposed for several applications including communication through biological fluids [31].

The CDs were excited at 405 nm and exhibited a broad emission spectrum ranging from about 450 nm to 650 nm (Figure 3a). An additional sharper peak was visible near 470 nm. This peak was also present in the solvent and can be ascribed to Raman scattering from water [35]. The Raman scattering component was clearly visible in this experiment because the brightness of the CDs was relatively low compared to that of an organic dye like Alexa 488.

We performed FCS using two different spectral detection bands: detection band 1 contained only the CD fluorescence band whilst detection band 2 contained both the fluorescence band and the 470 nm peak. Thus, data collected with spectral band 2 contained a larger fraction of uncorrelated background. In both cases the background correction increased the amplitude of the ACF, with a visible effect already at the highest concentration of CDs (Figure 3b,c middle). The trend of the number of particles as a function of dilution (Figure 3b,c bottom) was closer to directly proportional after background correction (detection band 1, slope = 1.16 ± 0.04; detection band 2, slope = 1.02 ± 0.5) compared to raw data (detection band 1, slope = 0.23 ± 0.09; detection band 2, slope = −0.2 ± 0.1). Note that the correction has a stronger effect for data collected with spectral band 2 (Figure 3c middle).

### 3.3. FCS Using a SPAD Array Detector

Another type of background may originate from detector-related noise, such as hot pixels typically found in SPAD arrays used as detectors in image scanning microscopy (ISM) setups. It has been recently shown that the use of SPAD arrays can further enhance the combination of confocal microscopy and fluctuation spectroscopy [36]. Here, we performed FCS using the 7 × 7 SPAD detector array (49 pixels total). In SPAD array detectors, hot pixels appear as individual elements exhibiting abnormally high dark count rates. To identify the hot pixels, we acquired a measurement of the dark counts. Hot pixels are detected as those pixels whose dark count rate (>20,000 counts/s) significantly deviates from the median value of dark counts in the array (~250 counts/s). In our PRISM system, the hot pixels are in position 20 and 44 (Figure 4a). Figure 4b compares the intensity counts of a hot pixel (n. 20, red) with a standard pixel (n. 24, black).

We evaluated the diffusion of PARPi-FL, a BODIPY FL-labeled olaparib (Figure 4c), at two concentrations (1 μM and 1 nM) in aqueous solution at room temperature. Single-point FCS measurements are performed on an ISM-based confocal microscope with a pixel dwell time of 10 μs.

We compared four data processing conditions: (i) raw data, i.e., the data as acquired by the SPAD array; (ii) background-corrected data, i.e., the raw data after background subtraction (background estimated from an identical measurement on the unlabeled sample); (iii) raw data excluding the two hot pixels, i.e., the data as acquired by the SPAD array excluding the signal of the two hot pixels from the FCS analysis; (iv) background-corrected data excluding the hot pixels, i.e., raw data excluding the two hot pixels after background subtraction (background estimated from an identical measurement on the unlabeled sample).

Figure 4d shows the ACFs of PARPi-FL at a higher concentration obtained from raw data including all 49 pixels (blue squares), background-corrected data (red circles), raw data excluding the two hot pixels (green triangles), and background-corrected data excluding the hot pixels (black triangles). As shown, the curves largely overlap at high concentration, where any type of background is negligible with respect to the fluorescence signal from PARPi-FL. The same analysis was carried out on 1 nM PARPi-FL in solution. In this case, raw data were strongly affected by background, and the curves improved progressively with background correction, hot pixel removal, and the combination of both (Figure 4e). Figure 4f shows the number of particles as a function of concentration. Log-log scale linear fits of the different conditions yielded slope values of 0.40 ± 0.08, 0.80 ± 0.12, 0.89 ± 0.11, and 0.97 ± 0.16, respectively, demonstrating that the trend closest to directly proportional is obtained when both hot pixels were removed and background correction was applied. Finally, Figure 4g shows that the diffusion coefficient remained largely constant in all cases, confirming that the determination of the diffusion constant is not affected by uncorrelated noise contributions.

## 4. Discussion

In this work we have shown that uncorrelated background of different origins can affect the determination of particle number by FCS. Thus, whenever the fraction of the background intensity with respect to the sample intensity is not negligible, the amplitude of the ACF should be corrected. Our method consists of subtracting the mean background intensity measured from an unlabeled sample. Other studies introduce additional free parameters into the fitting model to account for background contributions. Although powerful, these models increase the number of fit parameters and thus the risk of overfitting, especially when signal-to-noise ratios of the ACF are low. Lifetime gating is another efficient background isolation method, especially when there is a difference between the lifetime of the fluorophores and lifetime of the background signal. However, it requires lifetime detection or time-gating electronics, which are not available in all FCS setups.

In the case of a bright fluorophore, our correction becomes relevant only when the concentration of the fluorophore is below a given range. According to our measurements of Alexa 488 performed on the Leica SP8 confocal laser scanning microscope, this concentration range is in the order of ~10 nM.

Next, we have shown that background should be taken into account when FCS is used to count the number of fluorescent carbon dots in the observation volume. Another approach for nanoparticle counting is nanoparticle tracking analysis (NTA); however, this is limited in terms of nanoparticle diameter, which must generally be higher than 10 nm, and single particles with low fluorescence intensity are not efficiently detected in favor of agglomerates. Alternatively, nanoparticles could be counted via mass determination methods, like mass spectroscopy, Rutherford backscattering spectrometry (RBS) [37], or inductively coupled plasma mass spectrometry (ICP-MS) [38]. However, these approaches are expensive in terms of both cost and time, generally requiring specific and destructive sample preparation.

Finally, we have shown that the correction can be useful for FCS data acquired in an ISM setup equipped with a SPAD detector array. ISM setups can be seen as a technological evolution of confocal laser scanning microscopes and are now available from different microscope manufacturers [39,40]. In the context of FCS, ISM setups are attractive as they expand the capability of the confocal microscope to performing fluorescence fluctuation analysis in a single measurement [36,41]. In this respect, we believe that it is important to estimate the background of the detector and take it into account in the FCS analysis.

We note that our protocol can be applied only when the background contribution is independent from the concentration of fluorophores. Examples of this type of background are Raman scattering, detector noise, and autofluorescence. Background due to Raman scattering originates from the solvent and is dependent on the intensity of the excitation light. Different types of detectors (e.g., photomultipliers and SPAD arrays) will generate different levels of background due to their dark counts.

In contrast, our protocol cannot be applied when the background originates from the same fluorophores that generate the fluctuations (e.g., diffusion of unbound ligands in TIRF-FCS). In fact, in these cases, the background cannot be determined via a measurement on the unlabeled sample. Another challenging condition that we have not tested is the background generated by cellular or tissue autofluorescence. In fact, even if the level of autofluorescence background could be determined on an unlabeled sample, this type of background is non-homogeneous (spatially and temporally). Finally, if the background is correlated or partially correlated (i.e., it generates correlations that superimpose in the ACF), the entire shape of the ACF (not just its amplitude) will be distorted and our protocol cannot be applied.

## 5. Conclusions

In summary, we described a method to correct the effect of uncorrelated background in FCS analysis. The method can be used to perform accurate determination of particle number thanks to the subtraction of the mean background intensity measured from an unlabeled sample. The proposed protocol is relevant to researchers in biology, biophysics, or material sciences using FCS to estimate the concentration of fluorescent molecules or nanoparticles. Whenever the fraction of the background intensity with respect to the sample intensity is not negligible, correction of background is necessary for a more accurate determination of concentration. We believe that this new tool will be useful to estimate the background and take it into account in FCS analysis. Moreover, we believe that our correction procedure could have broader relevance for a wide range of FCS-related techniques in which background contributions can bias quantitative readouts. Methods such as FCCS, RICS, and other fluctuation spectroscopy approaches can benefit from a similar strategy for quantifying and compensating background signals.

## Figures and Tables

**Figure 1:**
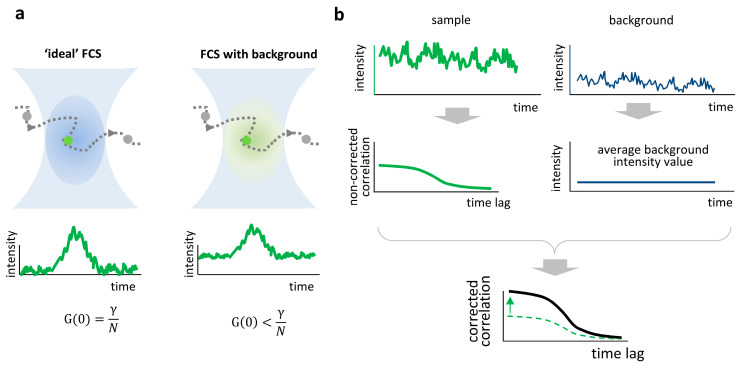
Background effect and correction in FCS. (**a**) Ideal FCS (**left**) assumes that only fluorescent molecules inside the confocal volume contribute to the signal, leading to a correlation amplitude proportional to the inverse of the average number of molecules N. In the presence of background signal (**right**), the correlation amplitude decreases, resulting in an overestimated particle number. (**b**) Schematic representation of background correction. The sample (green trace) and background (blue trace) intensity time traces are recorded. The average value of background intensity is used to correct the amplitude of the correlation function (black line, corrected; dashed green line, non-corrected).

**Figure 3:**
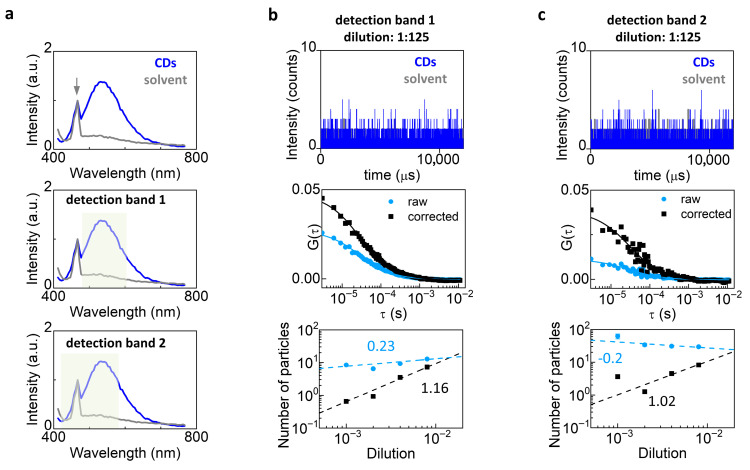
Background correction in FCS measurements of dim nanoparticles. (**a**) Emission spectra measured from the sample of carbon dots (CDs) (blue) and from the water solvent (gray). The arrow indicates the Raman peak of water. In the middle and bottom panels, the plot is duplicated and the two detection bands used for FCS measurements are indicated. (**b**,**c**) FCS of CDs at dilution 1:125 using detection band 1 (**b**) and detection band 2 (**c**). Shown are the intensity trace (**top**), the ACFs before (light blue) and after (black) background correction (**middle**), and a log-log plot of the number of particles before (light blue) and after (black) background correction versus dilution, with the corresponding apparent linear fits (**bottom**). To check for direct proportionality, we plotted the number of particles vs. dilution data in log-log scale and performed an apparent linear fit using Origin. In theory, direct proportionality should correspond to a slope equal to 1.

**Figure 4:**
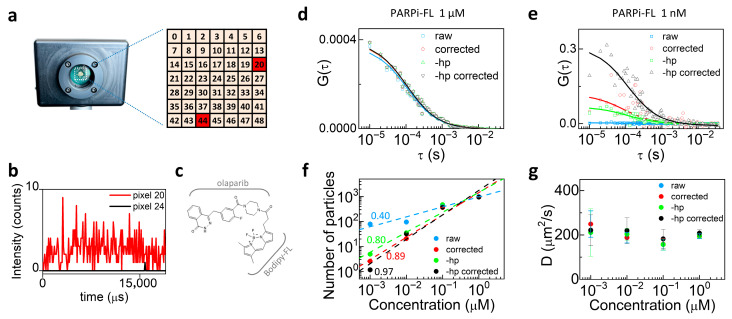
Background correction in SPAD array FCS measurements of PARPi-FL. (**a**) Scheme of the 7 × 7 SPAD array; hot pixels n. 20 and 44 are highlighted. (**b**) Intensity counts of a hot pixel (n. 20, red) compared with a normal pixel (n. 24, black). (**c**) Schematic of the molecule used in the experiment: PARPi-FL is a labeled drug composed of a fluorescent BODIPY-FL moiety and the PARP inhibitor olaparib. (**d**) ACFs of PARPi-FL in aqueous solution at 1 μM: raw data including all pixels (blue squares), background-corrected raw data (red circles), raw data excluding hot pixels (green triangles), and background-corrected raw data excluding hot pixels (black triangles). (**e**) ACFs of PARPi-FL in aqueous solution at 1 nM: background correction and hot pixel removal increasingly improve curve quality. (**f**) Log-log plots of the number of particles vs. concentration; to check for direct proportionality, we plotted the data in log-log scale and performed an apparent linear fit using Origin. In theory, direct proportionality should correspond to a slope equal to 1; apparent linear fits yield slopes = 0.40, 0.80, 0.89, and 0.97 for raw, background-corrected, hot-pixel removed, and background-corrected hot-pixel removed data, respectively. (**g**) Diffusion coefficients remain constant across all conditions.

## Data Availability

Data is contained within the article.
